# Observations and Projections of Heat Waves in South America

**DOI:** 10.1038/s41598-019-44614-4

**Published:** 2019-06-03

**Authors:** S. Feron, R. R. Cordero, A. Damiani, P. J. Llanillo, J. Jorquera, E. Sepulveda, V. Asencio, D. Laroze, F. Labbe, J. Carrasco, G. Torres

**Affiliations:** 1 Universidad de Santiago de Chile, Av. Bernardo O’Higgins 3363, Santiago, Chile; 2 Department of Earth System Science, Stanford University, Stanford, CA 94305–2210 United States of America; 3 Center for Environmental Remote Sensing, Chiba University, Chiba, Japan; 4 Instituto de Alta Investigación, Universidad de Tarapacá, Casilla 7D, Arica, Chile; 5 Universidad Técnica Federico Santa María, Av. Espana 1680, Valparaíso, Chile; 6 Universidad de Magallanes, Av. Bulnes 01855, Punta Arenas, Chile; 7 Direccion Meteorologica de Chile, Av. Portales 3450, Santiago, Chile

**Keywords:** Climate and Earth system modelling, Projection and prediction

## Abstract

Although Heat Waves (HWs) are expected to increase due to global warming, they are a regional phenomenon that demands for local analyses. In this paper, we assess four HW metrics (HW duration, HW frequency, HW amplitude, and number of HWs per season) as well as the share of extremely warm days (TX95, according to the 95th percentile) in South America (SA). Our analysis included observations as well as simulations from global and regional models. In particular, Regional Climate Models (RCMs) from the Coordinated Regional Climate Downscaling Experiment (CORDEX), and Global Climate Models (GCMs) from the Coupled Model Intercomparison Project Phase 5 (CMIP5) were used to project both TX95 estimates and HW metrics according to two representative concentration pathways (RCP4.5 and RCP8.5). We found that in recent decades the share of extremely warm days has at least doubled over the period December–January–February (DJF) in northern SA; less significant increases have been observed in southern SA. We also found that by midcentury, under the RCP4.5 scenario, extremely warm DJF days (as well as the number of HWs per season) are expected to increase by 5–10 times at locations close to the Equator and in the Atacama Desert. Increases are expected to be less pronounced in southern SA. Projections under the RCP8.5 scenario are more striking, particularly in tropical areas where half or more of the days could be extremely warm by midcentury.

## Introduction

While there is no standard definition for Heat Waves (HWs), they can be understood as a period of consecutive days with considerably warmer than usual conditions for a specific region and time of the year^1–4^.

HWs are of great importance not only for ecosystems, but also for socioeconomic systems, since extreme temperatures may lead to heat strokes, blights or failure of crops, and may also have negative impacts on the water and electricity supply^5,6^. Indeed, weather and climate extremes already cause significant economic damage and harm many lives each year^7–10^.

There is evidence that HWs have increased and intensified over the last few decades, and climate projections suggest further intensification in many regions^11–14^. An increasing threat to human life from HWs seems almost inevitable since, even if drastic reductions in emission of greenhouse gases (GHG) are enforced, the percentage of the world’s population exposed to a dangerous heat and humidity combination (for at least 20 days a year) will increase from about 30% nowadays to around 48% by 2100^15^. Under less restrictive emission scenarios, the relative frequency of present-day dangerous heat and humidity combinations could rise in the tropics and parts of the mid-latitudes by a factor of 100–250 by 2080^16^.

At a global scale, the study of the characteristics (intensity, frequency and duration) of future HWs has been addressed by using simulated scenarios from the Coupled Model Inter-comparison Project Phase 5 (CMIP5). Perkins-Kirkpatrick & Gibson^17^ found an increase in HW days (between 4–34 extra days per season) per degree Celsius of global warming. Limiting global warming to the lower Paris Agreement target of 1.5 °C brings along substantial positive effects and benefits^18^. In fact, stabilizing climate warming at 1.5 °C would decrease extreme heat-related mortality by 15–22% per summer in key European cities compared with stabilization at 2 °C^19^. Similarly, if warming is limited to 1.5 °C instead of 2 °C, the probability of HW events like those in southern Africa (1991/1992) and in northern Africa (2009/2010) could be reduced by about 25% and 20%, respectively^20^. Still, even if global warming is limited to 1.5 °C, a significant increase in the magnitude of HWs is expected to occur over Africa, South America and Southeast Asia^21^.

Several modeling and observational studies have been devoted to the analysis of HWs in the Northern Hemisphere. Specifically in the U.S.^22–24^, China^25,26^, South Korea^27^, South Asia^28^, as well as in Central and and Southern Europe^29–33^. Although less attention has been paid to HWs in the Southern Hemisphere, relevant efforts have targeted Australia^34–36^ and South America (SA)^37–42^.

Previous studies based on observations have shown that, in line with the global trend, cold (warm) extremes have decreased (increased) in SA over the period 1950–2010^38^. Significant regional differences have been found in the observed trends^39,40^. Ceccherini *et al*.^41^ reported a significant increase in the observed intensity and frequency of HWs in most of the megacities in SA since 1980. Piticar^42^ reported similar results for several Chilean locations since 1961. In general, observations agree with model simulations pointing toward an increase in the number of HWs by 2100 in northern SA^43^.

Building on these prior efforts, in this study we have assessed the progression and expected changes in four HW metrics (HW duration, HW frequency, HW amplitude, and number of HWs per season), as well as the share of extremely warm days over the period December-January-February (DJF). We considered as extremely warm days those that exceeded the 95th percentile of the daily maximum temperature anomaly distribution over a base period, while a HW was defined as a time span of at least 3 consecutive extremely warm days.

Our analysis included quality-controlled long-term temperature records from nine weather stations, simulations from CMIP5 Global Climate Models (GCMs)^44^, and estimates from Regional Climate Models (RCMs) from the Coordinated Regional Climate Downscaling Experiment (CORDEX)^45^, forced with GCMs. Both CMIP5 and CORDEX simulations were used to assess the expected progression of both extremely warm DJF days (TX95) and HW metrics according to two representative concentration pathways (RCP): RCP4.5 and RCP8.5.

We found that in recent decades the share of extremely warm DJF days has at least doubled in northern SA; less significant increases have been observed in southern SA. We also found that by midcentury, under the RCP4.5 scenario, extremely warm DJF days (as well as the number of HWs per season) are expected to increase 5–10 times at locations close to the Equator and in the Atacama Desert. Increments are expected to be less pronounced in southern SA. Projections under the RCP8.5 scenario point toward even larger disruptions in the HWs metrics, particularly in tropical areas. Methodological details are provided below.

## Data and Methods

In this study a HW is defined as a period of at least 3 consecutive days with the daily maximum temperatures (T_MAX_) exceeding a defined threshold during the considered season (DJF). The threshold was calculated at each location (or grid point of a climate model) by using the 95th percentile of the daily T_MAX_ anomaly distribution over a base period. The 95th percentile was used to account for more severe events since prior efforts have shown that extremely warm days (those exceeding the 95th percentile of the daily maximum temperature) can substantially increase mortality and morbidity rates^9,10^.

The Expert Team on Climate Change Detection and Indices (ETCCDI) provides 27 indices^46^, of which 16 are temperature indices that can be employed for characterizing HWs. In this paper, we used four HW metrics:

HW amplitude **(HWA)**: T_MAX_ anomaly (in °C) of the hottest day of any HW during a season;

HW duration (**HWD)**: the length (in days) of the longest HW during a season;

HW frequency **(HWF)**: number of HW days per season; and the

Number of HWs per season **(HWN**).

Note that according to the convention used by Cowan *et al*.^35^, HWD must be ≥3 days and is defined as a missing value in seasons without a heat wave. Likewise, HWA is defined as a missing value for seasons without a heat wave. However, for such seasons, HWF is defined as zero, and thus averaging over time can result in HWF <3 days”.

Following the methodology employed in prior studies^3,35^, we computed the base climatology for each DJF day using a centered 15-day window (i.e., we used 15-day rolling window of the T_MAX_ data from which the 95^th^ percentile was obtained each year) over the base period.

The histogram or the corresponding probability density function (PDF) of the daily T_MAX_ anomalies (the departure of daily T_MAX_ from the daily base climatology) allowed us to compute:

**TXM**: the average of the T_MAX_ anomaly (comparing TXM values computed over different periods allowed us in turn to assess the shift in T_MAX_ anomalies);

**STD**: the standard deviations (STDs) of T_MAX_ anomalies (comparing STD values computed over different periods allowed us in turn to assess the changes in variability of T_MAX_ anomalies);

**TX95**: share of extremely warm DJF days (i.e. the percentage of DJF days exceeding the 95th percentile of the T_MAX_ anomaly distribution corresponding to the base period).

In this study, we analyzed the daily T_MAX_ data rendered by Regional Climate Models (RCMs) from the Coordinated Regional Climate Downscaling Experiment (CORDEX)^45^. We used all the RCMs available for SAM-44 (South America) from CORDEX (except for the HadGEM2-ES model, which was excluded from the study as it has a different temporal resolution). In particular, we used the SMHI-RCA4 model^47^ provided by the Swedish Meteorological and Hydrological Institute Rossby Centre (SMHI), as well as the REMO2009 model^48^ developed at the Max-Planck-Institute for Meteorology. All of the CORDEX models have a spatial resolution of 0.44° x 0.44°.

The boundary conditions for the SMHI-RCA4 model were obtained from 8 GCMs from the Coupled Model Intercomparison Project 5 (CMIP5)^44^: (1) MPI-M-MPI-ESM-LR^49^; (2) CCCma-CanESM2^50^; (3) CSIRO-QCCCE-CSIRO-Mk3-6-0^51^; (4) IPSL-IPSL-CM5A-MR^52^; (5) MIROC-MIROC5^53^; (6) NOAA-GFDL-GFDL-ESM2M^54^; (7) NCC-NorESM1-M^55^; and (8) ICHEC-EC-EARTH^56^, while in case of the REMO2009 model, boundary conditions were also obtained from (1) MPI-M-MPI-ESM-LR^49^ Note that the latter GCM (MPI-M-MPI-ESM-LR) provided boundary conditions to both the SMHI-RCA4 model and the REMO2009 model.

The daily T_MAX_ data provided by the CMIP5 GCMs mentioned above were also analyzed. For consistency, we used the ensembles of the GCMs (see Table 1) that drove the RCMs considered in this study. However, we also explored the effects on our GCM-based results of selecting different ensemble members. In particular, we compared TX95 estimates computed over the period 2046–2055 by using simulations rendered by several ensemble members corresponding to the following GCMs: EC-EARTH (9 ensembles), CSIRO-Mk3-6-0 (9 ensembles), and CanESM2 (5 ensembles). We did not find significant differences between TX95 estimates based on different ensemble members (see Figs S1–S3), which suggests that the selection of a specific ensemble member does not substantially affect our outcomes.

**Table 1:** Global Climate Models (GCMs) considered in this study.

Institute	Full model acronym	Resolution	Main reference
Max Planck Institute for Meteorology	MPI-ESM-LR	1.9° × 1.9°	Giorgetta *et al*.^49^
Canadian Centre for Climate Modelling and Analysis	CanESM2	2.8° × 2.8°	Yang & Oleg^50^
CSIRO-QCCCE-CSIRO-Mk3-6-0	CSIRO-Mk3.6.0	1.9° × 1.9°	Rotstayn *et al*.^51^
Institut Pierre Simon Laplace	IPSL-CM5A-MR	1.3° × 2.5°	Dufresne *et al*.^52^
Atmosphere and Ocean Research Institute (The University of Tokyo), National Institute for Environmental Studies, and Japan Agency for Marine-Earth Science and Technology	MIROC5	1.4° × 1.4°	Watanabe *et al*.^53^
Geophysical Fluid Dynamics Laboratory	GFDL ESM2M	2.0° × 2.5°	Dunne *et al*.^54^
Norwegian Climate Centre	NorESM1-M	1.9° × 2.5°	Bentsen *et al*.^55^
Swedish Meteorological and Hydrological Institute, Rossby Centre	ICHEC-EC-EARTH	1.1° × 1.1°	Koenigk *et al*.^56^

Following prior efforts^57,58^, in this study our projections are based on the multi model mean (MMM) of HW metrics and of TX95 values, previously computed for each selected model separately (either GCM or RCM). Moreover, note that although we used a fair number of GCMs, we also explored the possibility of including additional GCMs. In this regard, the MMM computed by using the GCMs in Table 1 was compared with the MMM obtained by adding other available GCMs. We did not find significant changes in the MMM.

**Figure 1:**
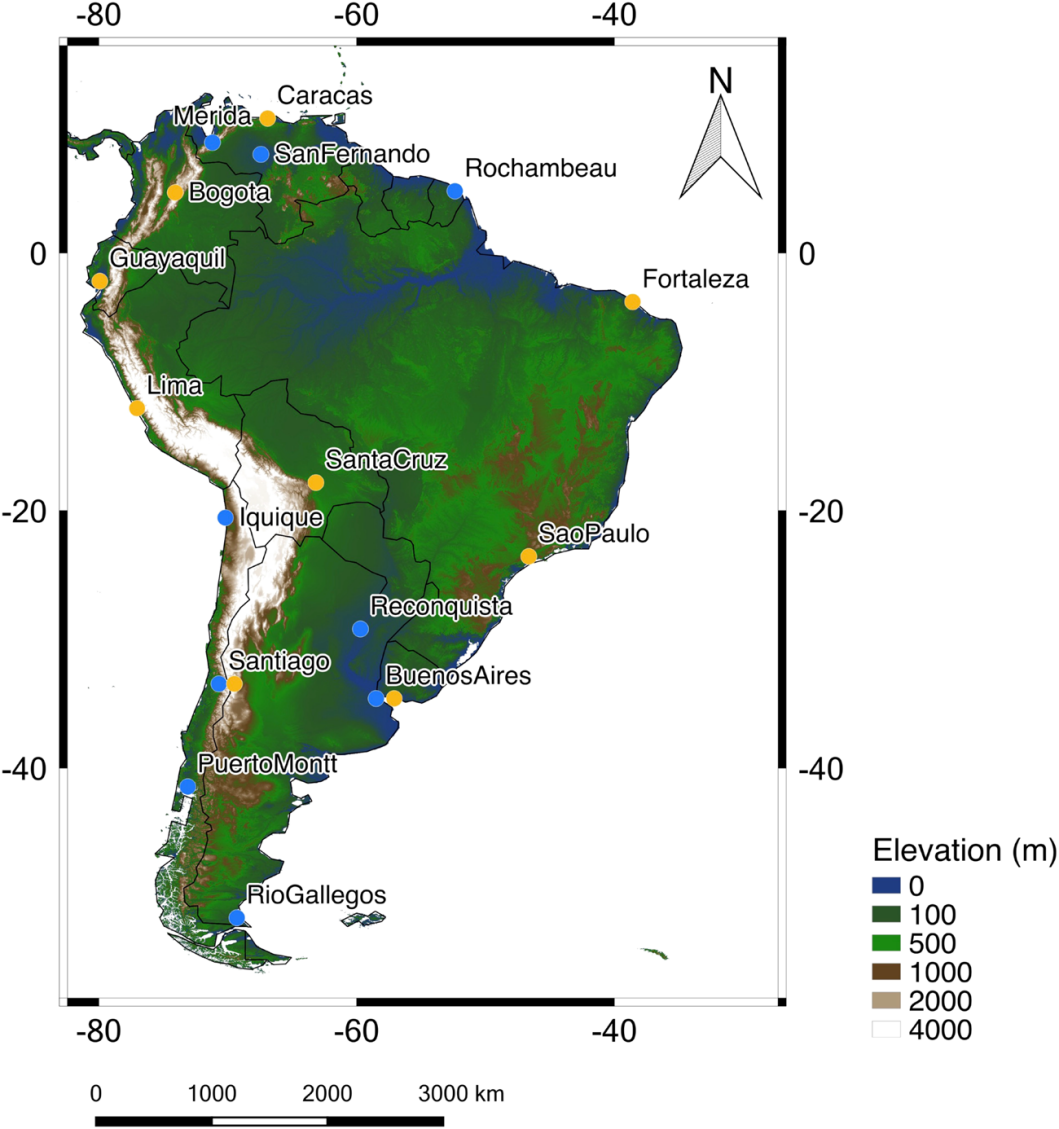
Locations of weather stations considered in this study (see blue dots). RCM simulations were used to project TX95 values and HW metrics for future climate scenarios (RCP4.5 and RCP 8.5) for 9 major cities (see yellow dots). The elevation map was created using the CGIAR-CSI SRTM dataset^91^. Plot was generated by using Python’s Matplotlib Library^92^.

Aimed at further comparisons, quality-controlled T_MAX_ observations were obtained from several weather stations provided by the National Oceanic and Atmospheric Administration (NOAA) (see https://www.ncdc.noaa.gov/cdo-web/ ^59^). Only stations with at least 75% of the data available (from the 1st of January 1961 to the 31st of December 2016) were selected. Weather stations abiding by this criterion are maintained in Chile, Argentina, Venezuela and French Guiana (see blue dots in Fig. 1). Since these locations correspond to urban zones, coastal locations, or areas of complicated topography, they can be hardly representative of the regional surroundings.

In addition, we compared TX95 estimates computed from the following reanalysis datasets: the ERA-Interim (ERAI) reanalysis^60^, the NCEP-DOE Reanalysis 2 dataset^61^, and the Climate Prediction Center (CPC) Global Daily Temperature dataset^62^. TX95 estimates computed from these 3 datasets over the period 2006–2015 are shown in Fig. S4. Based on the significant differences between these estimates, we avoided using reanalysis datasets as reference for model evaluations in SA.

In this study, the HW metrics and TX95 values were computed considering the base period 1961–1990, except in the case of comparisons between reanalysis datasets within which the base period 1979–2005 was used (both ERAI and NCEP Reanalysis 2 are only available since 1979).

## Results and Discussion

### Observations

**Figure 2:**
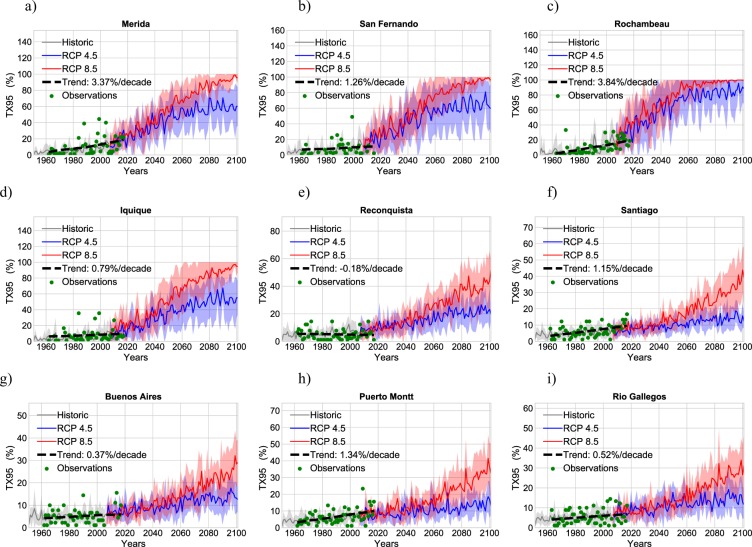
Share of extremely warm DJF days (TX95) from observations over the period 1961–2016 (green dots), from historical RCM simulations over the period 1951–2005 (gray line), and from RCM projections over the period 2005–2100 (blue line for RCP4.5; red line for RCP8.5). TX95 values correspond to the percentage of DJF days exceeding the 95th percentile of the T_MAX_ anomaly distribution corresponding to the base period. The trend line (as well as the decadal trend computed using observations over the period 1961–2016) is also shown in each plot. (**a**) Merida; (**b**) San Fernando; (**c**) Rochambeau; (**d**) Iquique; (**e**) Reconquista; (**f**) Santiago; (**g**) Buenos Aires; (**h**) Puerto Montt; (**i**) Rio Gallegos. Plots were generated by using Python’s Matplotlib Library^92^.

Figure 2 depicts the share of extremely warm DJF days (TX95) at nine locations, ranging from latitude 9°N (Mérida) to latitude 52°S (Rio Gallegos). TX95 values were computed: from quality-controlled temperature records over the period 1961–2016 (green dots), from historical RCM simulations over the period 1951–2005 (gray line), and from RCM projections over the period 2006–2100 (blue line for RCP4.5; red line for RCP8.5). In the case of the RCM-based estimates, thick lines indicate the multi model mean of TX95 estimates, while shadows stand for the dispersion bounds (based on the standard deviation computed by using the multi model spread of TX95 estimates).

Each plot in Fig. 2 includes a trend line (as well as the decadal trend) for the observations over the period 1961–2016. As shown in Fig. 2, TX95 values computed from observations exhibit an increasing trend especially in northern SA (see Fig. 2a–c). TX95 values from observations at latitudes higher than 20°S (see Fig. 2d–i) exhibit a less strong trend (particularly in the case of coastal locations in the Atacama Desert; see Fig. 2d).

As a reference, in the supplementary material (see Fig. S4) we have included the TX95 estimates over the period 2006–2015, computed from the 3 reanalysis datasets (CPC, NCEP-DOE Reanalysis 2, and ERAI). The TX95 estimates from different datasets exhibit significant differences: TX95 values from NCEP-DOE Reanalysis 2 (see Fig. S4b) are lower in the eastern Amazon region and in southern SA than estimates from both CPC and ERAI datasets (see Fig. S4a and c). Significant differences were also found in the case of the Atacama Desert (northern Chile/southern Peru) where ERAI-based TX95 estimates (see Fig. S4c) were significantly higher that estimates based on other reanalysis datasets. The differences between TX95 estimates computed from different reanalysis datasets make unwise to use them for further comparisons.

### RCMs

RCM simulations were used to reconstruct the TX95 progression over the period 1951–2005.

The quality-controlled temperature records allowed us to test these historical RCM simulations. In general over the period 1961–2005, we found a good agreement between TX95 values computed from observations (green dots in Fig. 2) and from historical RCM simulations (grey curves in Fig. 2). This agreement becomes apparent when comparing decadal averages as shown in Fig. S5. An exception was detected in the case of Reconquista (northern Argentina; see Fig. 2e and Fig. S5e), where TX95 values computed from observations have been significantly below RCM-based estimates in recent decades.

RCM simulations were also used to project TX95 estimates according to two representative concentration pathways (RCP4.5 and RCP8.5). RCM-based estimates over the period 2006–2100 in Fig. 2 show that the share of extremely warm days is expected to increase considerably, especially close to the equator (see Fig. 2a–c). According to the RCP4.5 scenario, TX95 estimates will reach about 50% by midcentury in Mérida and San Fernando and even higher values in Rochambeau (see blue lines in Fig. 2a–c). Iquique (in the Atacama Desert) is below these values with a TX95 estimate of about 35% under the RCP4.5 scenario by midcentury (see blue line in Fig. 2d). Increments in extremely warm days are significantly lower at higher latitudes under the RCP4.5 scenario such that by midcentury, TX95 values lower than 15% are expected in the case of Reconquista, Santiago, Buenos Aires, Puerto Montt, and Rio Gallegos (see blue lines in Fig. 2e–i).

As expected, TX95 projections under the RCP8.5 scenario show larger increases. By the end of the century, TX95 estimates are projected to reach almost 100% in northern SA (see red lines in Fig. 2a–c), and more than 80% in the Atacama Desert (Iquique) (see red line in Fig. 2d). The number of extremely warm days would also increase at higher latitudes under the RCP8.5 scenario but less significantly; TX95 estimates are expected to range from 20% to 40% by the end of the century in the case of locations at latitudes higher than 20°S (Reconquista, Santiago, Buenos Aires, Puerto Montt, and Rio Gallegos) (see red lines in Fig. 2e–i).

### HW Projections

**Figure 3:**
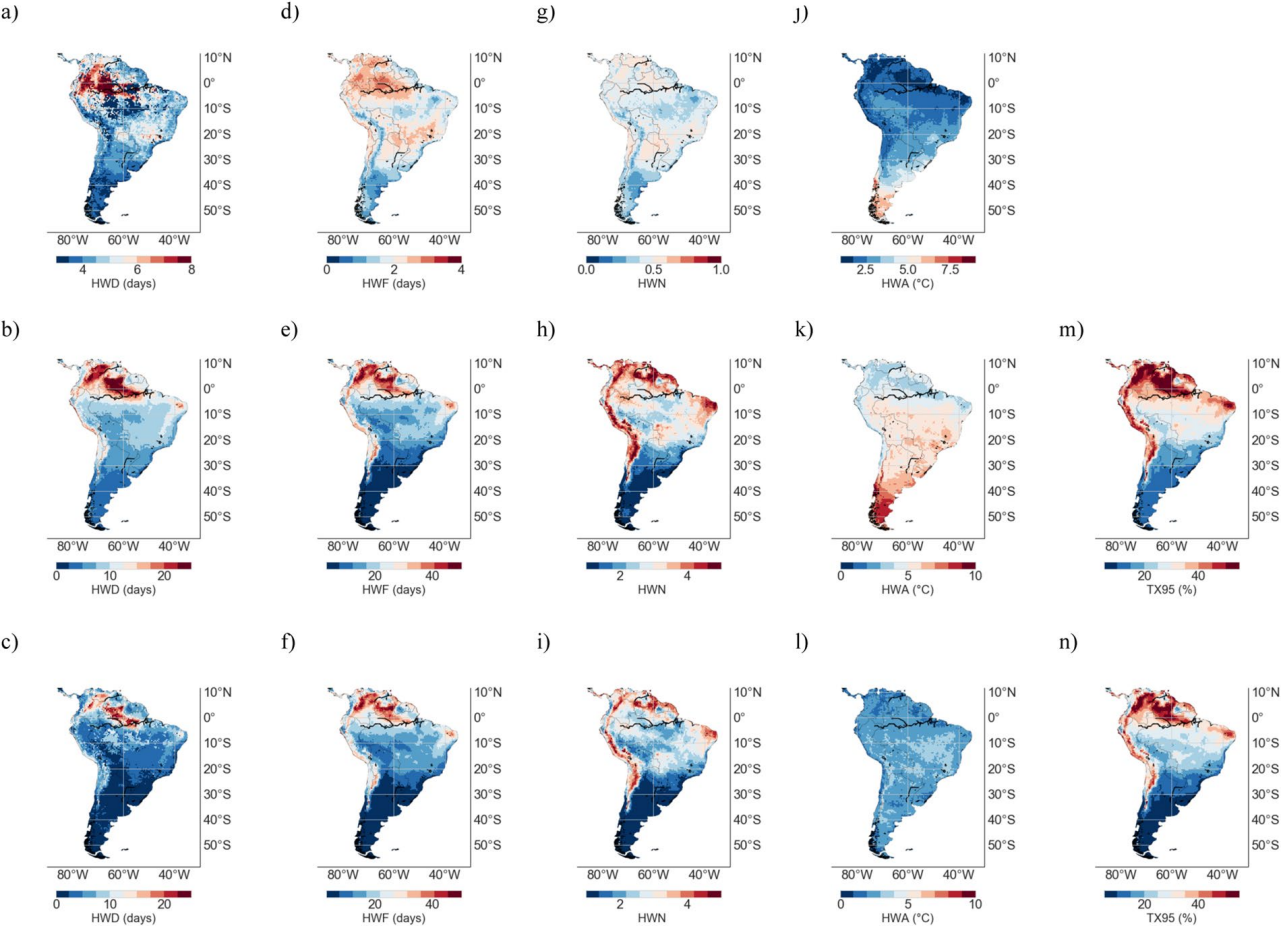
Multi-Model-Mean (MMM) of HW metrics from RCM simulations over the period 1961–1990 (1^st^ row); TX95 estimates over the base period 1961–1990 are not shown since they were by definition 5%. MMM of HW metrics and TX95 estimates from RCM simulations over the period 2046–2055 under the RCP4.5 scenario (2^nd^ row). Change, 1961–1990 to 2046–2055 (RCP4.5), in TX95 estimates and in HW metrics (3^nd^ row). (**a**–**c**) HWD; (**d**–**f**) HWF; (**g**–**i**) HWN; (**j**–**l**) HWA; and (**m**,**n**) TX95. Plots were generated by using Python’s Matplotlib Library^92^.

RCM and GCM simulations were exploited in order to project HW metrics in SA according to the representative concentration pathway RCP4.5. Figure 3 shows the MMM of HW metrics computed from RCM simulations over the base period 1961–1990 (1^st^ row), as well as the MMM of HW metrics and TX95 estimates from RCM simulations over the period 2046–2055 under the RCP4.5 scenario (2^nd^ row). Figure 3 also shows the change, 1961–1990 to 2046–2055 (RCP4.5), in TX95 estimates and in HW metrics (3^rd^ row).

The projections in Fig. 3 show that TX95 estimates and all HW metrics would exhibit significant increments by mid century. HWs are expected to be more frequent and last longer, especially in northern SA (including the northern Amazon region) as well as in the Atacama Desert. In these areas, the extremely warm DJF days (TX95) are projected to increase by 5–10 times (from 5% in 1961–1990 to 25–50% in 2046–2055), while the HW days per season (HWF**)** are expected to rise from less than 3 days in 1961–1990 to 15–30 days in 2046–2055.

The increments in TX95 estimates and HW metrics shown in Fig. 3 are less pronounced in southern SA. Extremely warm DJF days in southern Patagonia are expected to increase from 5% in 1961–1990 to about 10% in 2046–2055, while the HW days per season (HWF**)** are expected to rise from about 1 days in 1961–1990 to about 5 days in 2046–2055. HWF estimates are expected to remain below 15 days per season in Southern Brazil, Uruguay, Paraguay, and Argentina.

HWN projections exhibit similar regional features as both HWF and TX95 estimates. Under the RCP4.5 scenario, by mid-century, the number of HWs is expected to at least double in southern SA, while they may increase 5–10 times at locations in the Atacama Desert and along the coastline of northern SA. These figures depict a significantly severer scenario than in central Europe, for example, where by mid-century HWN estimates (according to the 90th percentile) are expected to double in summer under the RCP4.5 scenario^33^.

By mid-century and under the RCP4.5 scenario, the number of DJF HWs is expected to range from less that 2 in southern SA to more than 4 in northern SA and the Atacama Desert. Estimates in central SA and southern SA are comparable to those reported for Australia^34^ and for South Korea^27^, respectively. In South Korea, the number of summer HWs (according to the 90th percentile) is expected to be greater that 3 (5) for the RCP4.5 (RCP8.5) over the period 2081–2100^27^, while in Australia, under the A2 scenario (comparable to the RCP8.5 scenario), summer HWN estimates (according to the 90th percentile) are projected to be about 2 in central Australia and about 3 in northern Australia over the period 2020–2039^34^.

HWA estimates exhibit different regional features than other HW metrics. Figure 3 shows that the T_MAX_ anomaly of the hottest day of the HWs is expected to increase about 2 °C throughout SA. Unlike the changes in other HW metrics, the increments in HWA projected by midcentury do not exhibit clear regional features (although increments are somehow larger in Brazil and at high altitude Andean locations). Nevertheless, by midcentury, Patagonia (southern Argentina and Chile) is expected to undergo HW amplitudes of up to 10 °C, whereas HWA estimates in northern SA and the northern Amazon region are expected to remain below 6 °C.

**Figure 4:**
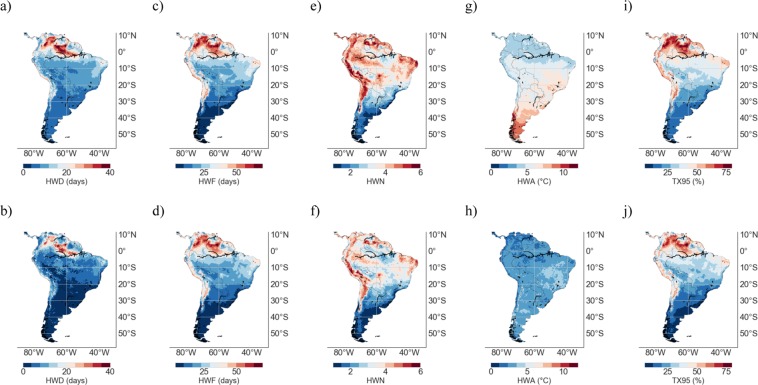
Multi-Model-Mean (MMM) of HW metrics and TX95 estimates from RCM simulations over the period 2090–2100 under the RCP4.5 scenario (1^st^ row). Change, 1961–1990 to 2090–2100 (RCP4.5), in HW metrics and in TX95 estimates (2^nd^ row). (**a**,**b**) HWD; (**c**,**d**) HWF; (**e**,**f**) HWN; (**g**,**h**) HWA; and (**i**,**j**) TX95. Plots were generated by using Python’s Matplotlib Library^92^.

Figure 4 shows the MMM of the TX95 estimates and of the HW metrics computed from RCM simulations over the period to 2091–2100 under the RCP4.5 scenario (1^st^ row), as well as the change, 1961–1990 to 2091–2100 (RCP4.5), in TX95 estimates and in the HW metrics (2^nd^ row). Compared with changes by mid century (see Fig. 3), projections are more striking by the end of the century. Indeed, Fig. 4 shows that even under the RCP4.5 scenario, half or more of the DJF days are expected to be extremely warm by the end of the century in northern SA and in the Atacama Desert. Although increases shown in Figs 3 and 4 exhibit very similar regional features, TX95 estimates and HW metrics in Fig. 4 are in general 25–50% higher than those in Fig. 3.

**Figure 5:**
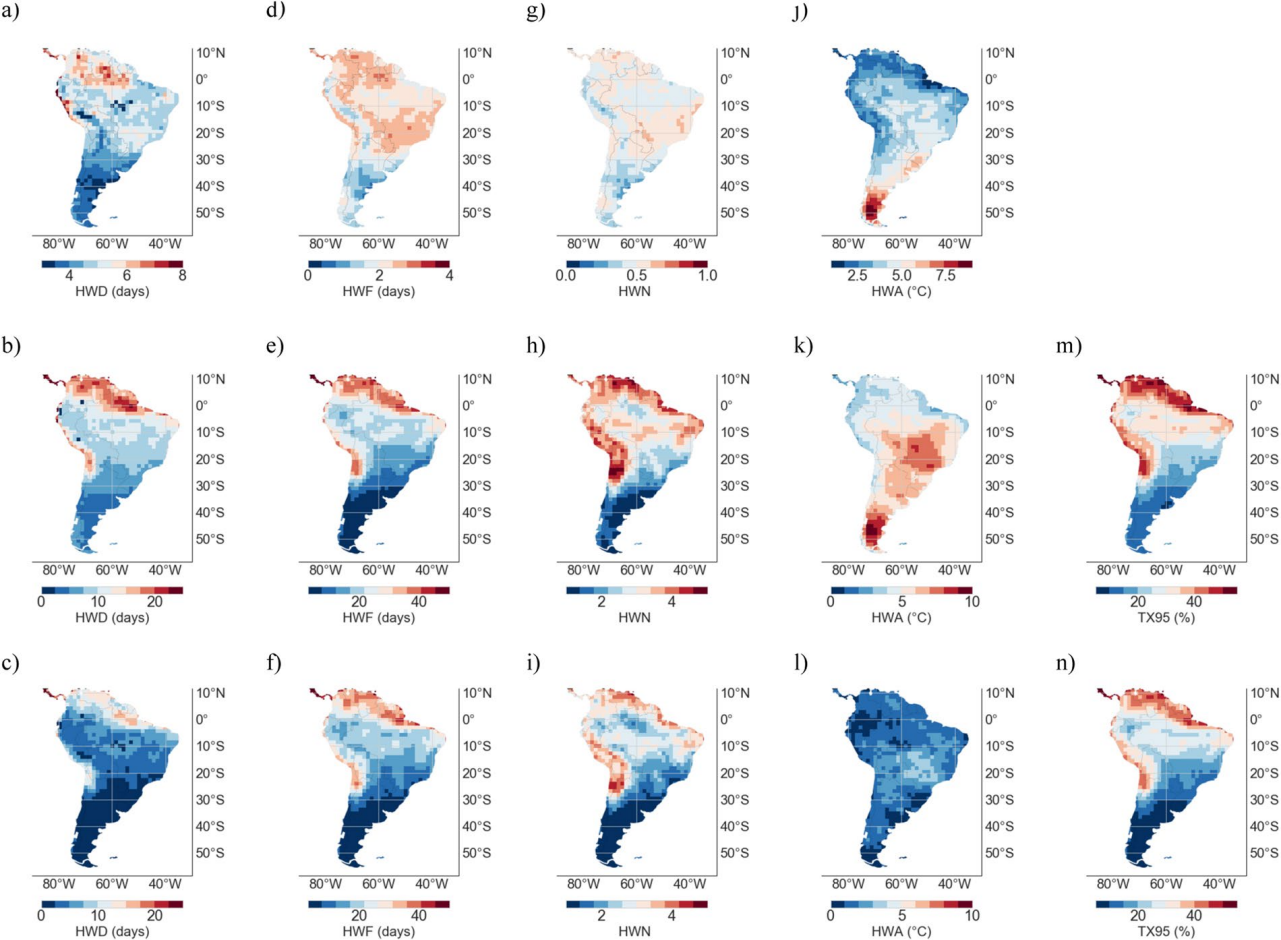
Multi-Model-Mean (MMM) of HW metrics from GCM simulations over the period 1961–1990 (1^st^ row); TX95 estimates over the base period 1961–1990 are not shown since they were by definition 5%. MMM of HW metrics and TX95 estimates from GCM simulations over the period 2046–2055 under the RCP4.5 scenario (2^nd^ row). Change, 1961–1990 to 2046–2055 (RCP4.5), in TX95 estimates and in HW metrics (3^nd^ row). (**a**–**c**) HWD; (**d**–**f**) HWF; (**g**–**i**) HWN; (**j**–**l**) HWA; and (**m**,**n**) TX95. Plots were generated by using Python’s Matplotlib Library^92^.

**Figure 6:**
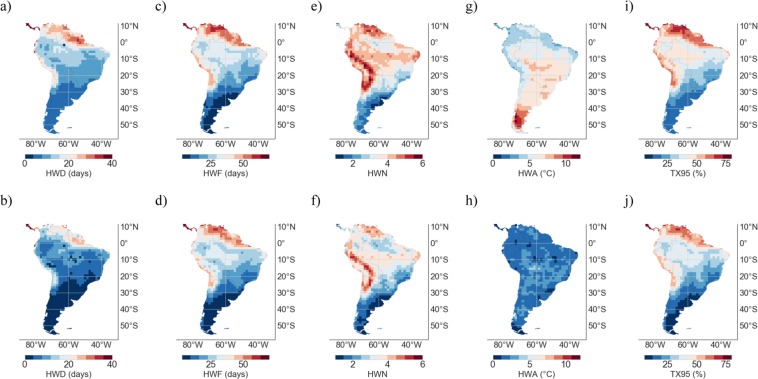
Multi-Model-Mean (MMM) of TX95 estimates and HW metrics computed from GCM simulations over the period 2090–2100 under the RCP4.5 scenario (1^st^ row). Change, 1961–1990 to 2090–2100 (RCP4.5), in TX95 estimates and in HW metrics (2^nd^ row). (**a**,**b**) HWD; (**c**,**d**) HWF; (**e**,**f**) HWN; (**g**,**h**) HWA; and (**i**,**j**) TX95. Plots were generated by using Python’s Matplotlib Library^92^.

It is worth comparing RCM-based simulations in Figs 3, 4 with projections based on GCMs. Figure 5 shows the MMM of HW metrics from GCM simulations over the base period 1961–1990 (1^st^ row), as well as the MMM of HW metrics and of TX95 estimates from GCM simulations over the period 2046–2055 under the RCP4.5 scenario (2^nd^ row). Figure 5 also shows the change, 1961–1990 to 2046–2055 (RCP4.5), in TX95 estimates and in the HW metrics from GCM simulations (3^rd^ row). Figure 6 shows the MMM of the TX95 estimates and of the HW metrics from GCM simulations over the period to 2091–2100 under the RCP4.5 scenario (1^st^ row), as well as the change, 1961–1990 to 2091–2100 (RCP4.5), in TX95 estimates and in the HW metrics from GCM simulations (2^nd^ row).

TX95 estimates and HW metrics, computed from GCM simulations under the RCP4.5 scenario over the period 2046–2055 (see Fig. 5) and over the period 2091–2100 (see Fig. 6), exhibit similar regional features as the corresponding RCM-based estimates (see Figs 3 and 4, respectively). These similarities were expected since, as indicated above, plots in Figs 5, 6 were computed by using the same GCMs that provided the boundary conditions to the RCMs used to compute plots in Figs 3, 4. In other words, Figs 5, 6 are based on the GCMs that drove the RCMs used in Figs 3, 4.

Nevertheless, some differences are apparent between RCM-based estimates and the corresponding GCM-based estimates, especially in coastal zones and in areas of complicated topography; in these areas, Figs 3, 4 show lower values for TX95, HWD, HWF, and HWN, than the corresponding Figs 5, 6. Part of these differences may be related to the resolution of GCMs; the lower resolution of GCMs (with respect to RCM estimates) has been found to be problematic in complex terrain regions^63–65^.

The differences between RCM-based and GCM-based estimates are also apparent in Fig. S5; for example, in the case of Santiago (see Fig. S5f; a major Andean city), TX95 estimates computed from RCM simulations are significantly below GCM simulations. These differences were expected since prior efforts^65^ have found (in areas of complicated topography) significant differences between RCM and GCM simulations in SA. In direct contrast to the case of Santiago, differences between RCM-based and GCM-based estimates are minor in the cases of Rochambeau (see Fig. S5c) and Buenos Aires (see Fig. S5g).

The differences shown in Fig. S5 between RCMs and GCMs may also be influenced by the variability of the simulated data. For example, a smaller variability leads to narrower frequency distributions of the daily maximum temperatures, which renders more extreme warm days than wider frequency distributions under similar increases in the mean temperature. As shown elsewhere^66^, the effect of the variability can lead to differences between RCM-based and GCM-based estimates, and may explain why RCM-based projections appear to be biased cold with respect to GCM-based projections in Fig. S5. In our case, GCMs simulated narrower frequency distributions than RCMs’, making GCM-based TX95 estimates higher than the corresponding RCM-based TX95 estimates. Still, with the exception of Santiago (see Fig. S5f; where the topography likely plays a dominant role), the differences between RCM-based and GCM-based TX95 estimates shown in Fig. S5 become significant only after mid-century.

### Extreme Temperatures in major cities

**Figure 7:**
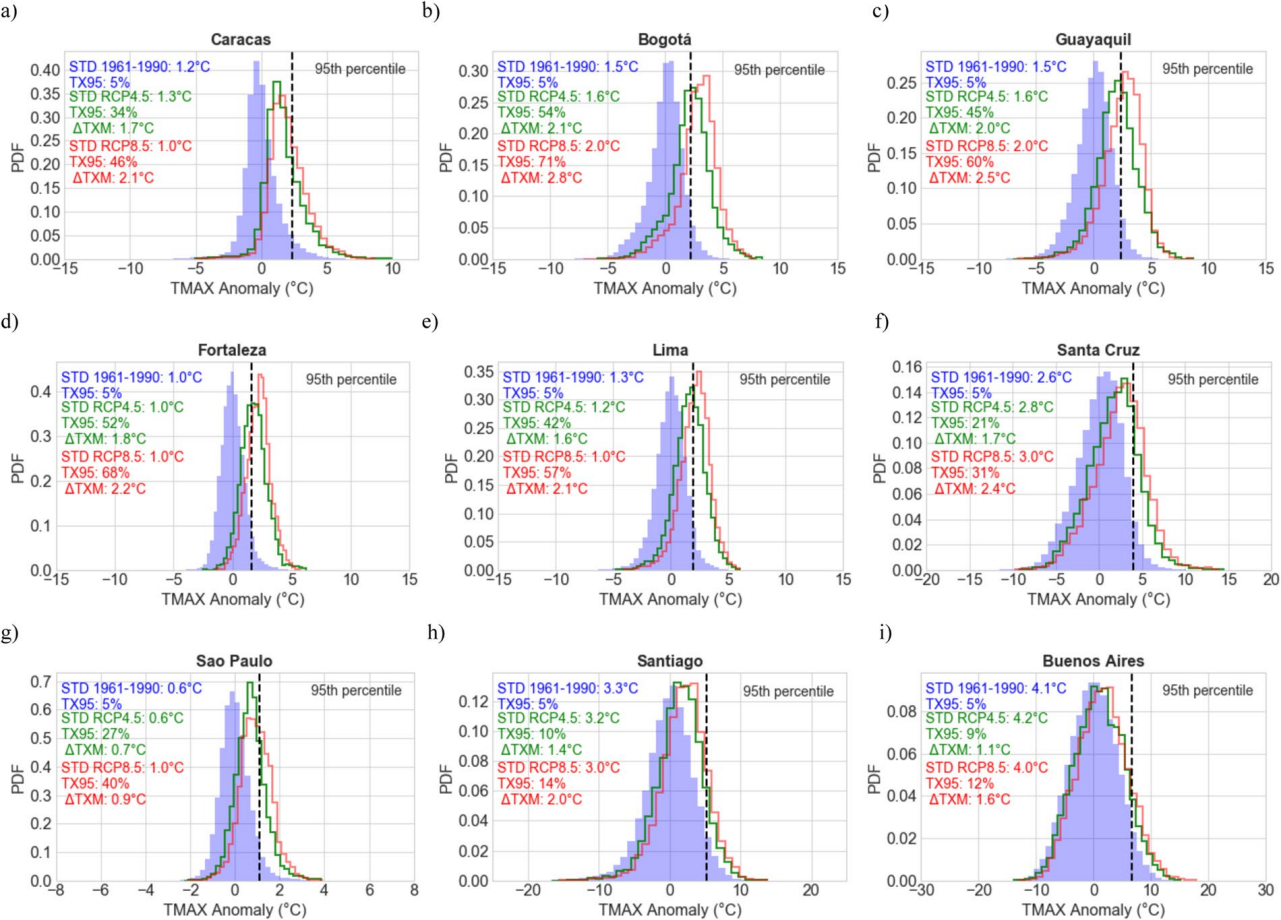
Histograms of the daily DJF T_MAX_ anomalies rendered by the RCMs over the base period 1961–1990 (blue histograms) as well as over the period 2046–2055 (green histograms for RCP 4.5; red histograms for RCP8.5). (**a**) Caracas; (**b**) Bogotá; (**c**) Guayaquil; (**d**) Fortaleza; (**e**) Lima; (**f**) Santa Cruz; (**g**) Sao Paulo; (**h**) Santiago; (**i**) Buenos Aires. The dotted vertical line in each plot indicates the 95th percentile of the T_MAX_ anomaly distribution corresponding to the base period. Also shown in the upper left corner of each plot and for each histogram: the standard deviation (STD); the share of extremely warm DJF days (TX95); and the shift in the mean (TXM), 1961–1990 to 2046–2055, of the DJF T_MAX_ anomalies. Plots were generated by using Python’s Matplotlib Library^92^.

We further analyzed the changes in HWs projected for most populated cities (see yellow dots in Fig. 1) in SA^67^: Sao Paulo 21.2 million; Lima 12.1 million; Bogota 10.2 million; Fortaleza 4 million; Santiago 6.1 million; Santa Cruz 1.4 million; Buenos Aires 13 million; and Caracas 2.9 million inhabitants. Figure 7 shows the histograms of the DJF daily T_MAX_ anomalies in these selected cities, rendered from RCM simulations over the base period 1961–1990 (blue histograms), as well as over the period 2046–2055 (see green histograms for RCP 4.5; see red histograms for RCP8.5).

The dotted vertical line in each plot of Fig. 7 indicates the 95th percentile of the DJF T_MAX_ anomaly distribution corresponding to the base period 1961–1990. The interception of this vertical line with the histograms corresponding to the period 2046–2055 allows assessing the increases in extremely warm DJF days (TX95) projected by mid century.

Under the RCP4.5 scenario, extremely warm DJF days (TX95) are expected to increase by about 10 times (from 5% in 1961–1990 to above 50% in 2046–2055) in Bogota (see Fig. 7b) and Fortaleza (see Fig. 7d); while in Caracas (see Fig. 7a), Guayaquil (see Fig. 7c) and Lima (see Fig. 7e), TX95 estimates are expected to rise about 7–9 times (from 5% in 1961–1990 to about 34–45% in 2046–2055). This is in good agreement with Figs 3–5 where, under the RCP 4.5 scenario, TX95 estimates computed from RCM simulations are expected to increase 5–10 times by mid century at locations close to the Equator.

As discussed in the prior section, the increase in extremely warm DJF days at higher latitudes under the RCP4.5 scenario is projected to be less significant than in the tropics. In this regard, TX95 estimates are projected to increase from 5% in 1961–1990 to 10% in Santiago (see Fig. 7h) and to 9% in Buenos Aires (see Fig. 7i) by midcentury.

As expected, the increments in the TX95 estimates and the HW metrics are higher under the RCP 8.5 scenario than under the RCP4.5 scenario. However, there are significant regional differences in these increments. The percentage of extremely warm DJF days is only slightly higher in absolute terms for RCP8.5 than for RCP4.5 at latitudes higher than 30°S. For example, TX95 estimates in Santiago are 14% for RCP8.5 and 10% for RCP4.5 by mid century (see Fig. 7h); TX95 estimates in Buenos Aires for RCP8.5 and for RCP4.5 are 12% and 9%, respectively (see Fig. 7i). The differences in TX95 estimates between RCP8.5 and RCP4.5 are substantially greater in absolute terms in tropical areas. For example, in Caracas TX95 is 46% for RCP8.5 and 34% for RCP4.5 (see Fig. 7a), in Bogotá TX95 is 71% for RCP8.5 and 54% for RCP4.5 (see Fig. 7b), in Guayaquil (Fortaleza) TX95 is 60% (68%) for RCP8.5 and 45% (52%) for RCP4.5 (see Fig. 7c–d), and in Lima TX95 is 57% for RCP8.5 and 42% for RCP4.5 (see Fig. 7e).

The regional differences in the expected TX95 increases (substantially larger in the tropics than at higher latitudes) are consistent with previous studies^17,21,34^. Most of these regional differences result from the fact that the variability of the T_MAX_ anomalies (i.e. the STD value) tends to be smaller in the tropics than at higher latitudes. STDs of the T_MAX_ anomalies are depicted at the upper left corner of each plot in Fig. 7. These STD values confirm that the variability of the T_MAX_ anomalies is significantly higher in southern SA (Santiago or Buenos Aires) than in the tropics (Sao Paulo or Caracas). This result is consistent with prior efforts that have found a relatively small variability at low latitude locations^68,69^. A smaller variability leads to narrower frequency distributions of the T_MAX_ anomalies, which renders more extreme warm days than wider frequency distributions under similar increases in the mean temperature. As shown in the histograms in Fig. 7, although the shifts in the mean (TXM) were comparable, increases in the share of extremely warm days (TX95) are substantially larger in the case of tropical locations (such as Caracas or Sao Paulo) than in the case of mid-latitude locations (such as Santiago or Buenos Aires). Indeed, in tropical regions, where the present-day variability and the seasonal cycle are small, even a moderate mean temperature increase results in more heat waves^21^.

In contrast to prior studies focused on Europe^70,71^,model simulations do not project significant changes in the variability of T_MAX_ anomalies in the upcoming decades in SA. As shown in Fig. 7, regardless of the locations in SA, there are no great differences between STD values corresponding to the base period 1961–1990 and STD values corresponding to the period 2046–2055 (for both RCP4.5 and RCP8.5). Therefore, the role of the variability in the expected TX95 increases is likely minor.

The increases in the extremely warm DJF days (TX95) expected in SA by midcentury are driven by shifts in the mean (TXM). This result is consistent with previous efforts^72^ that found that the mean temperature shift (ΔTXM) is the dominant factor (compared to a substantially weaker effect of the variability) for the increasing occurrence of hot extremes in many regions of the world. The shifts (ΔTXM), from 1961–1990 to 2046–2055, are also shown in the upper left corner of each plot in Fig. 7. The significant ΔTXM values (for both RCP4.5 and RCP8.5) lead to the strong increases in TX95 estimates expected by midcentury, especially in cities close to the equator (Fig. 7a–e) where ΔTXM is higher than 1.5 °C for RCP4.5 and higher than 2 °C for RCP8.5. The shifts in the mean, 1961–1990 to 2046–2055, of the DJF T_MAX_ anomalies are projected to be less significant at latitudes higher than 20°S: ΔTXM estimates are lower than 1.5 °C for RCP4.5 and around 2 °C or lower for RCP8.5 at these locations.

The substantial differences between TX95 estimates in tropical areas projected under different scenarios (RCP8.5 and RCP4.5) underline the fact that curbing global warming will make a significant difference in the number of extremely warm DJF days that South American cities at low latitudes will have to endure.

A summary of expected changes in TX95 estimates and in HW metrics by mid-century (under the RCP4.5 scenario) for selected cities (including some of the population hubs in SA) can be found in supplementary material (see Table S1).

### Inter-model differences

**Figure 8:**
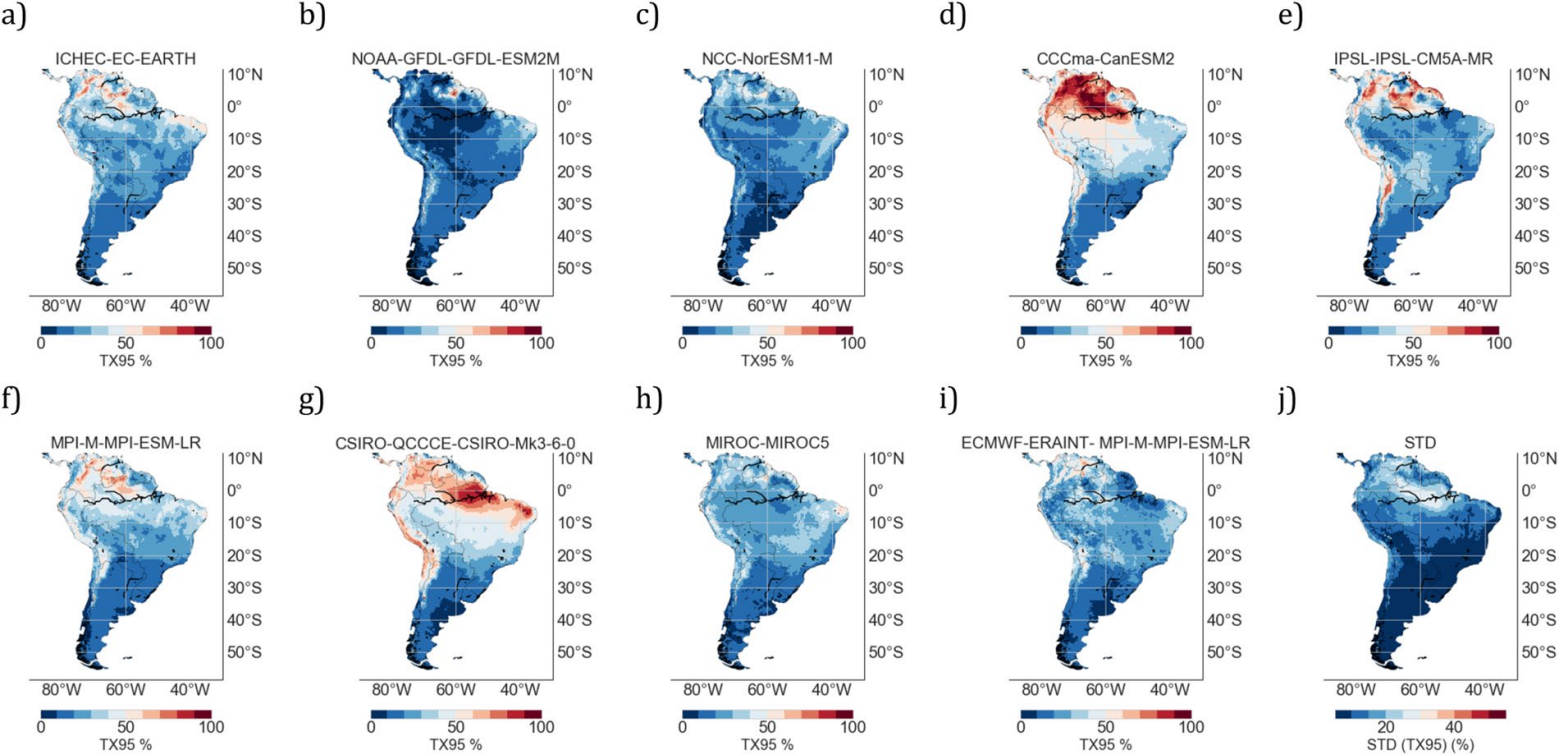
TX95 estimates by midcentury (2046–2055) computed from different RCMs: (**a**) ICHEC-EC-EARTH; (**b**) NOAA-GFDL-GFDL-ESM2M; (**c**) NCC-NorESM1-M; (**d**) CCCma-CanESM2; (**e**) IPSL-IPSL-CM5A-MR; (**f**) MPI-M-MPI-ESM-LR; (**g**) CSIRO-QCCCE-CSIRO-Mk3-6-0; (**h**) MIROC-MIROC5; (**i**) ECMWF-ERAINT- MPI-M-MPI-ESM-LR; (**j**) Standard deviation (STD) computed by using the spread of decadal TX95 estimates (2046–2055) from different RCMs (see plots **a**–**i**). In this figure, *RCMs in plot 8f and in 8i were driven by the same GCM* (*MPI*-*M*-*MPI*-*ESM*-*LR*; *see plot 9f*). Plots were generated by using Python’s Matplotlib Library^92^.

**Figure 9:**
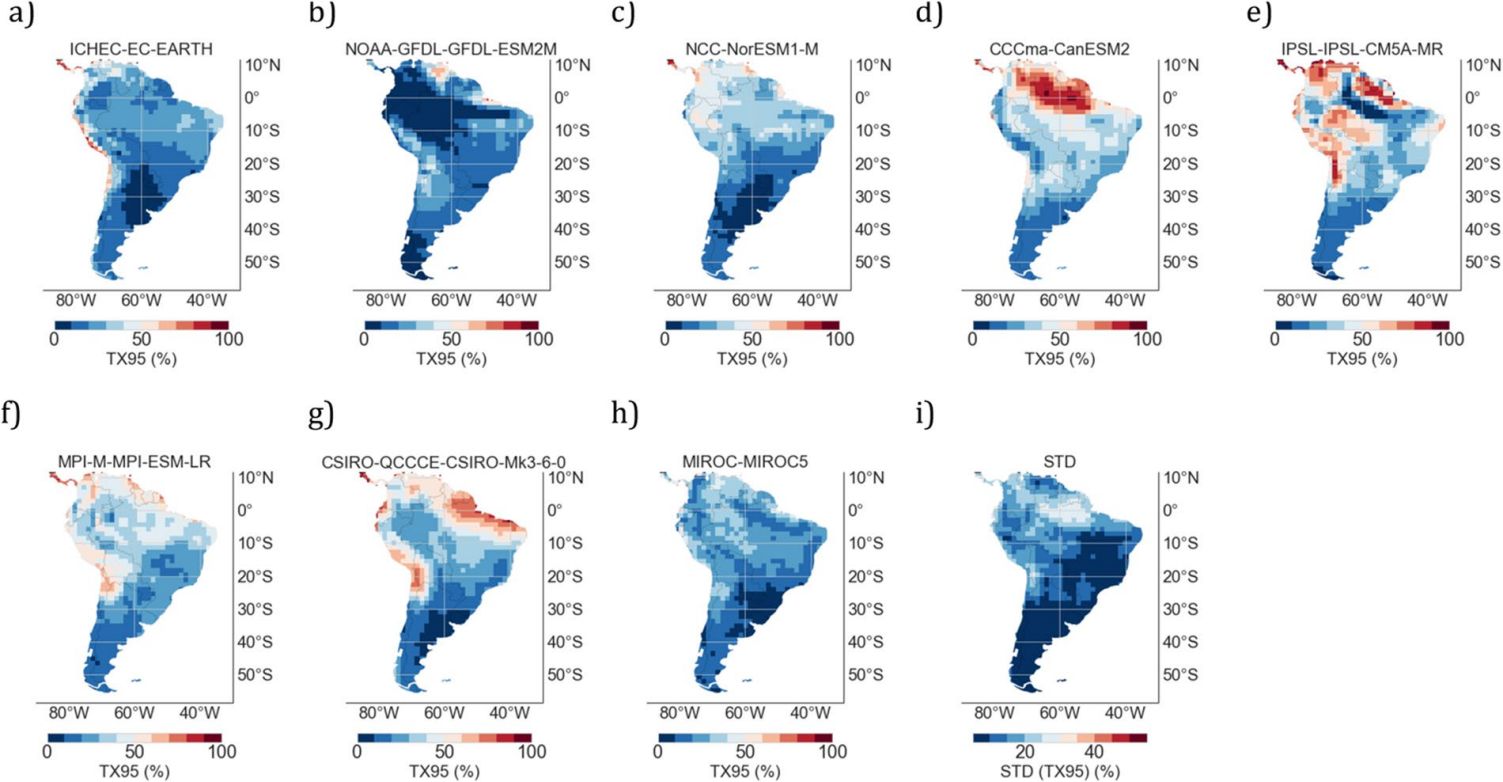
TX95 estimates by midcentury (2046–2055) computed from different GCMs: (**a**) ICHEC-EC-EARTH; (**b**) NOAA-GFDL-GFDL-ESM2M; (**c**) NCC-NorESM1-M; (**d**) CCCma-CanESM2; (**e**) IPSL-IPSL-CM5A-MR; (**f**) MPI-M-MPI-ESM-LR; (**g**) CSIRO-QCCCE-CSIRO-Mk3-6-0; (**h**) MIROC-MIROC5; (**i**) Standard deviation (STD) computed by using the spread of decadal TX95 estimates (2046–2055) from different GCMs (see plots **a**–**h**). Plots were generated by using Python’s Matplotlib Library^92^.

Our projections, based on the MMM of both HW metrics and TX95 values, may be affected by the selection of ensembles and models. We explored the effects of selecting different models on the results. In particular, we compared TX95 estimates computed under the RCP4.5 scenario over the period 2046–2055 by using 9 RCMs (see Fig. 8a–i) and by 8 GCMs (see Fig. 9a–h). Comparing Figs 8a and 9a (as well as Figs 8b and 9b and so on), we found in general terms a good agreement between RCMs and their corresponding GCMs (i.e. the GCM that drove the RCM). Similarly to our findings when comparing MMM values, significant differences between the RCM estimates and their corresponding GCMs were found at certain locations (for example, those surrounded by a complicated topography).

In agreement with prior efforts^36^, we did find significant inter-model differences in the case of RCMs (note the spread of TX95 predictions from different models in Fig. 8a–i) as well as in the case of GCMs (note the spread of TX95 predictions from different models in Fig. 9a–h), mainly in northern SA. The inter-model differences were characterized by computing the standard deviation (STD) of the spread of decadal TX95 estimates (2046–2055) from different models. Figure 8j shows the STD values of RCM-based TX95 estimates shown in Fig. 8a–i, while Fig. 9i shows the STD values of GCM-based TX95 estimates shown in Fig. 9a–h.

Relatively high STD values in Figs 8j and 9i (up to 25% in Venezuela, northern Brazil, and Colombia) indicate significant inter-model differences in the TX95 estimates in northern SA, where by mid century CanESM2 projects TX95 values of up to 80% (Fig. 8d), while NOAA-GFDL-GFDL-ESM2M (Fig. 8b) estimates TX95 values close to 20%. The CSIRO-Mk3-6-0 (Fig. 8g) also projects high TX95 values (around 70%) in northeastern Brazil. The lowest STD values in Figs 8j and 9i (less than 5% in Patagonia) indicate less inter-model differences in the TX95 estimates in southern SA. Similar regional features in inter-model differences are observed in the case of GCMs (see Fig. 9).

According to Strobach & Bel^73^, the differences between model outputs can be explained by internal variability (revealed by comparing realizations from different ensemble members from one model that contain different initial conditions), or by differences in the parameterization of individual models (i.e. the climate sensitivity), which usually translates into the spread of climate predictions from different models.

We explored the effects on our GCM-based results of selecting different ensemble members. In particular, we compared TX95 estimates computed over the period 2046–2055 by using different ensemble members corresponding to the following GCMs: EC-EARTH (9 ensembles), CSIRO-Mk3-6-0 (9 ensembles), and CanESM2 (5 ensembles). We did not find significant differences between TX95 estimates based on different ensemble members (see Figs S1–S3), which suggests that the selection of a specific ensemble member (i.e. the *internal variability*) has no substantial effect on our outcomes.

The inter-model differences shown in Fig. 9 are likely influenced by differences in the parameterization of individual models. Various climate centers have described some of the biases resulting from their parameterization^55,74,75^. For instance, the surface temperature from the EC-EARTH model is known to be too cold (with the exception of the Southern Ocean and parts of the extratropical Northern Hemisphere)^76^. Other studies have reported too warm (extreme) temperature estimates from the CSIRO-Mk3-6-0 and the CanESM2 models in Venezuela and northern Brazil, whereas NORESM and EC-EARTH underestimate the (maximum) surface temperature in most parts of SA^76,77^. If these biases shift the whole PDF of the T_MAX_ anomalies, they may not necessarily lead to biases in HW estimates. However, HW estimates may be affected in case of biases affecting daily maximum temperatures.

A different model parameterization (i.e. a different climate sensitivity) significantly affects projections on the intertropical convergence zone (ITCZ)^78,79^. For instance, while some models (e.g. MIROC5 and NCC-NorESM1) have a relatively small double ITCZ bias, other models (e.g. GFDL-ESM2M, CanESM2 or CSIRO-Mk3-6-0) have a more pronounced bias^80^. The movement of the ITCZ alters the cloud cover and humidity, and has been found to cause changes in the heat flux with T_MAX_ anomalies of up to 3.5 °C in northern-central Brazil^81^. A shown elsewhere^82^, a positive bias in precipitations may lead to an underestimation of T_MAX_ and vice versa. The negative correlation between precipitation and T_MAX_ has been found to be particularly strong for central SA^83^. Therefore, biases in GCMs stemming from variations in the ITCZ projections may partially explain the significant inter-model differences in HWs found in northern SA.

Although due to a lack of evaporative cooling, heat extremes can be amplified by dry conditions^84^, many GCMs have also shown an amplification of heat extremes by land surface dryness in wet regions, a finding not supported by observations^57^. This suggests that part of the inter-model differences shown in Fig. 9 (particularly in northern SA) may be related to biases in the simulation of the land-atmosphere coupling.

Since it may cancel out individual model biases (if models are not too strongly correlated^36,85^), in this study our projections are based on the MMM. Figures 3m and 5m show the MMM of TX95 estimates computed from RCM and GCM simulations, respectively, over the period 2046–2055 under the RCP4.5 scenario. It is worth comparing Figs 3m and 5m with Figs 8j and 9i, which show STD values of TX95 estimates (2046–2055) computed from the models used in this study. It can be observed in these figures that the expected increase in the share of extremely warm DJF days in northern SA (up to 50% in 2046–2055) is twice as high as the STD values also in northern SA (up to 25% in 2046–2055). In southern SA, the signal-to-noise ratio (MMM/STD) is similarly strong. Although they cannot mask the significant increases in TX95 estimates expected by mid-century, the inter-model differences shown in Figs 8 and 9 do affect the uncertainty of our projections.

Finally, although we used a fair number of GCMs, we also explored the possibility of including additional GCMs. In this regard, the MMM computed by using the selected GCMs was compared with the MMM obtained by adding other available GCMs. We did not find significant changes in the MMM.

## Summary and Conclusions

In this paper, quality-controlled long-term observations from nine weather stations in SA were used to assess four HW metrics (HWD, HWF, HWA and HWN), as well as the share of extremely warm DJF days (TX95). We found that the share of extremely warm DJF days has at least doubled in recent decades in northern SA; less significant increases were observed in southern SA.

Moreover, RCM and GCM simulations were exploited in order to project both TX95 estimates and HW metrics according to two representative concentration pathways (RCP4.5 and RCP8.5). Although both TX95 estimates and HW metrics were computed for each model separately (either GCM or RCM), our results are based on the multi model mean (MMM) of the HW metrics and the TX95 values. RCM and GCM simulations exhibited similar regional features, though some differences (likely related to the coarser resolution of GCMs) were apparent especially in coastal zones and in areas of complicated topography (such as the Andean Region).

Both RCM and GCM simulations show significant increments in extremely warm DJF days (TX95) and HW metrics by midcentury; HWs are expected to be more frequent and last longer, especially in northern SA (including the northern Amazon region) as well as along the pacific coastline of the Atacama Desert.

Under the RCP4.5 scenario, TX95 estimates in northern SA and the Atacama Desert are projected to increase by 5-10 times (from 5% in 1961–1990 to 25–50% in 2046–2055), while the HW days per season (HWF**)** are expected to rise from less than 3 days in 1961–1990 to 15–30 days in 2046–2055. The increments in TX95 estimates and HW metrics are less pronounced in southern SA. Extremely warm DJF days in southern Patagonia are expected to increase from 5% in 1961–1990 to about 10% in 2046–2055, while the HW days per season (HWF**)** are expected to rise from about 1 days in 1961–1990 to about 5 days in 2046–2055. HWF estimates are expected to remain below 15 days per season in Southern Brazil, Uruguay, Paraguay, and Argentina. Under the RCP4.5 scenario, by mid-century, the number of HWs per season (HWN) is expected to at least double in southern SA, while they may increase 5–10 times or more in the Atacama Desert and along the coastline of northern SA. Indeed, by mid-century HWN estimates are expected to range from less that 2 in southern SA to more than 3 in northern SA and the Atacama Desert.

TX95 projections under the RCP8.5 scenario show larger increases. By the end of the century, TX95 estimates are projected to reach almost 100% in northern SA and more than 80% in the Atacama Desert. The number of extremely warm days would also increase at higher latitudes under the RCP8.5 scenario but less significantly; TX95 estimates are expected to range from 20% to 40% by the end of the century in the case of locations at latitudes higher than 20°S.

Tropical major cities are expected to be strongly affected by HWs and daily record temperatures. In Caracas for example, TX95 estimates under the RCP4.5 scenario are projected to increase about 7 times (up to 34% in 2046–2055), while in Guayaquil, they are expected to rise up to 9 times (up to 45% in 2046–2055). Increases in the extremely warm DJF days in major cities in southern SA are projected to be less significant. In Buenos Aires and Santiago for example, under the RCP4.5 scenario, the share of extremely warm DJF days is expected to rise from 5% over the base period to 9–10% in 2046–2055.

As expected, projected changes under the RCP8.5 scenario are more severe. However, the extremely warm DJF days expected by mid century under RCP8.5 scenario are substantially greater in tropical areas than those projected under the RCP4.5 scenario. For example, in Caracas TX95 is 46% for RCP8.5 and 34% for RCP4.5, while in Guayaquil TX95 is 60% for RCP8.5 and 45% for RCP4.5. The number of extremely warm days will also increase at higher latitudes under the RCP8.5 scenario but less significantly. For example, by mid century in Santiago TX95 is 14% for RCP8.5 and 10% for RCP4.5, while in Buenos Aires TX95 is 12% for RCP8.5 and 9% for RCP4.5.

As other climate-related impacts, the expected increases in the share of extremely warm days would exacerbate existing global inequalities^86,87^ exposing vulnerable and disadvantaged populations (especially in northern SA) to further risks. Increases in HWs pose a serious challenge to countries in northern SA (all developing countries) due to their vulnerability (determined by population density, percentage of the poor population and their spatial distribution)^88^, as well as their limited adaptation capacity (determined by limited access to information/resources and a weak institutional framework /governance)^89,90^. These facts underline the importance of curbing GHG emissions, especially for countries in northern SA. Since there are substantial differences between TX95 estimates in tropical areas under different scenarios (RCP8.5 and RCP4.5), limiting global warming will make a significant difference in the number of extremely warm days that population at low latitudes will have to endure by mid century.

## Supplementary information

Suplementary material

## Data Availability

The datasets generated and analyzed during the current study are available from the corresponding author on reasonable request.
