# Supplementary material for: Observations and Projections of Heat Waves in South America
Source: Sci Rep. 2019 Jun 3;9:8173. doi: 10.1038/s41598-019-44614-4 (PMC6547650; doi:10.1038/s41598-019-44614-4)
Supplement: Supplementary file 1 — Suplementary material [file 41598_2019_44614_MOESM1_ESM.pdf]

Observations and Projections of Heat Waves in South America

S. Feron[1,2], R.R. Cordero[1,*], A. Damiani[1,3], P.J. Llanillo[1], J. Jorquera[1], E. Sepulveda[1], V. Asencio[1], D. Laroze[4], F. Labbe[5], J. Carrasco[6], G. Torres[7]

[1]Universidad de Santiago de Chile, Av. Bernardo O'Higgins 3363, Santiago, Chile
[2] Department of Earth System Science, Stanford University, Stanford, CA 94305–2210, United States of America
[3]Center for Environmental Remote Sensing, Chiba University, Chiba, Japan
[4]Instituto de Alta Investigación, Universidad de Tarapacá, Casilla 7D, Arica, Chile
[5] Universidad Técnica Federico Santa María, Av. Espana 1680, Valparaíso, Chile
[6]Universidad de Magallanes, Av. Bulnes 01855, Punta Arenas, Chile
[7]Direccion Meteorologica de Chile, Av. Portales 3450, Santiago, Chile

* Corresponding author
Email: raul.cordero@usach,cl
Tel: +56-9-89018916
Fax: +56-2-27181299

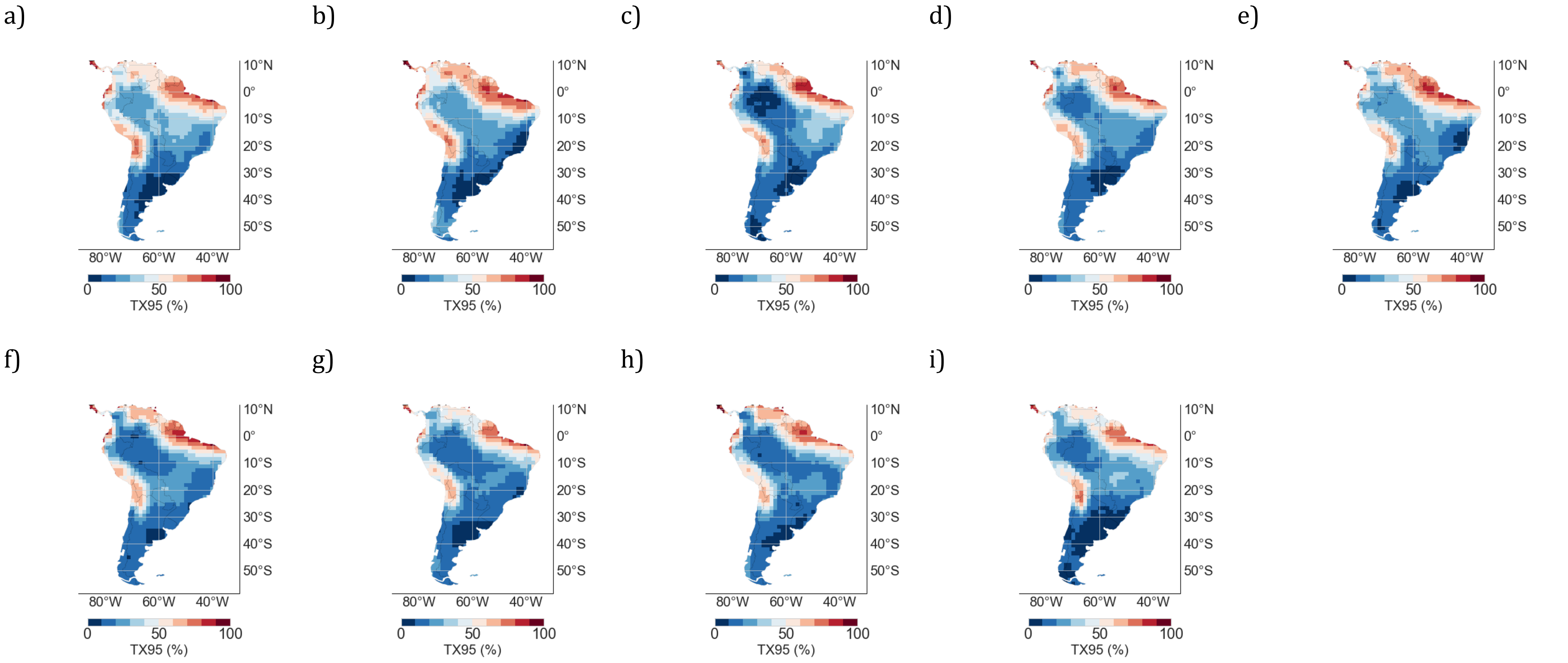

Fig. S1. TX95 estimates by midcentury (2046-2055) computed by using different ensemble members of the CSIRO-Mk3-6-0 model: a) r1i1p1; b) r2i1p1; c) r3i1p1; d) r4i1p1; e) r5i1p1; f) r6i1p1; g) r7i1p1; h) r8i2p1; i) r9i1p1. Plots were generated by using PYTHON's Matplotlib Library[92].

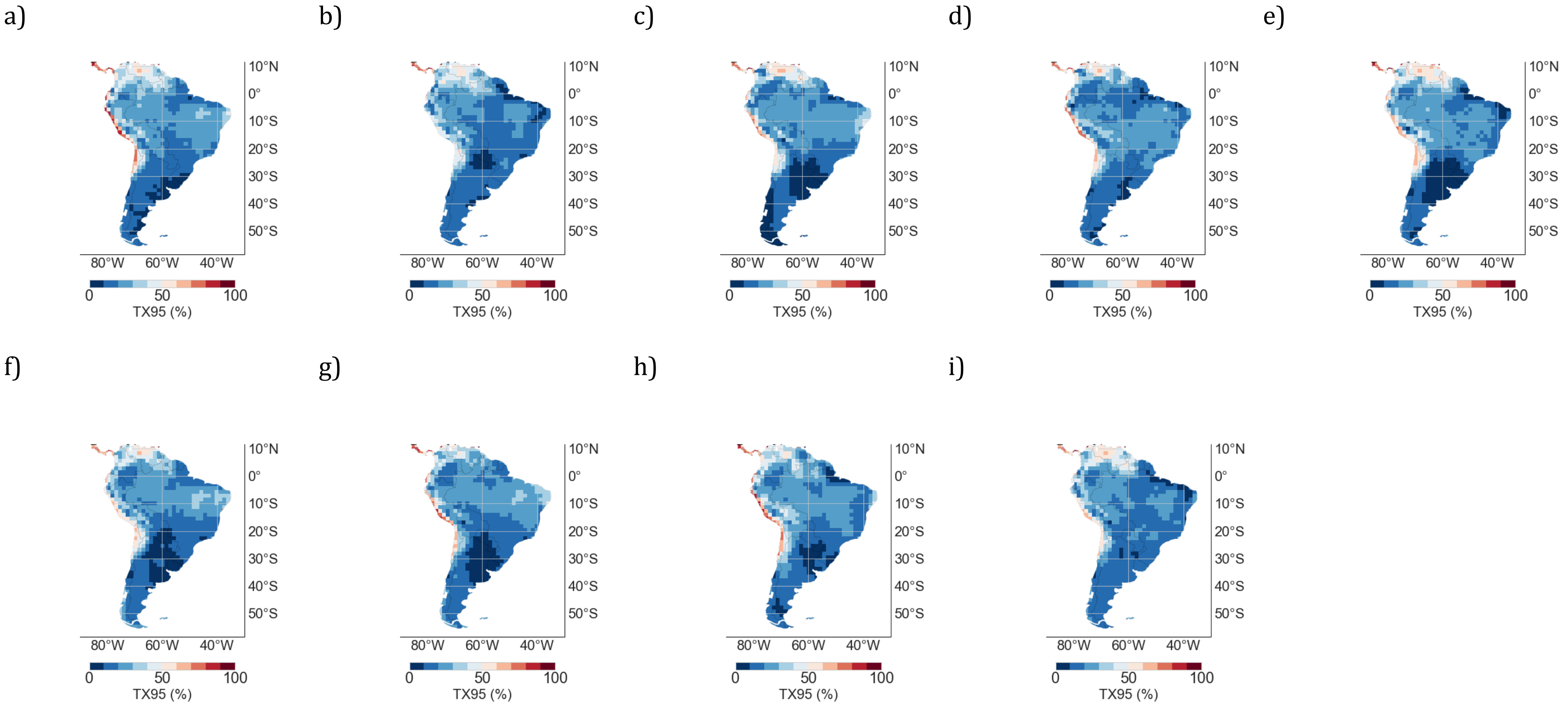

Fig. S2. TX95 estimates by midcentury (2046-2055) computed by using different ensemble members of the EC-EARTH model: a) r1i1p1; b) r2i1p1; c) r5i1p1; d) r7i1p1; e) r8i1p1; f) r11i1p1; g) r11i1p1; h) r11i2p1; i) r14i1p1. Plots were generated by using PYTHON's Matplotlib Library[92].

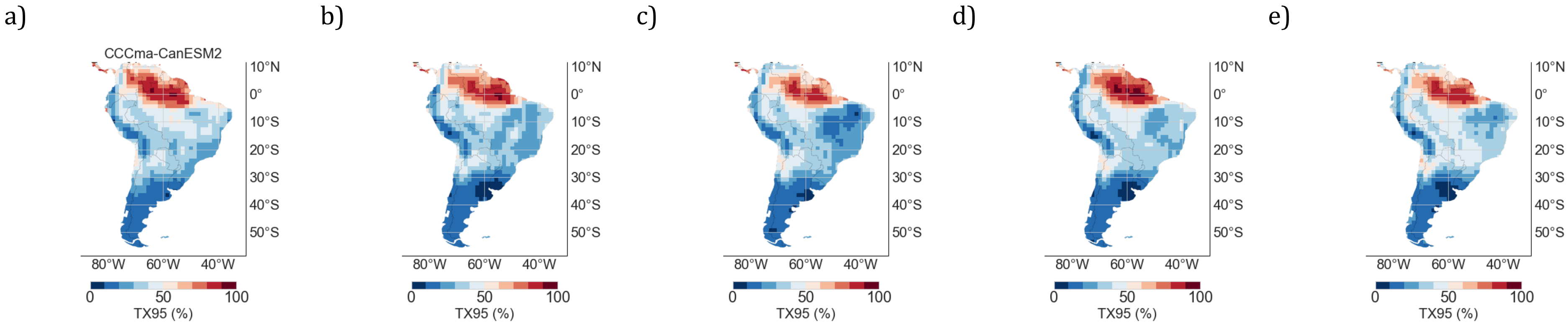

Fig. S3. TX95 estimates by midcentury (2046-2055) computed by using different ensemble members of the CanESM2 model: a) r1i1p1; b) r2i1p1; c) r3i1p1; d) r4i1p1; e) r5i1p1. Plots were generated by using PYTHON's Matplotlib Library[92].

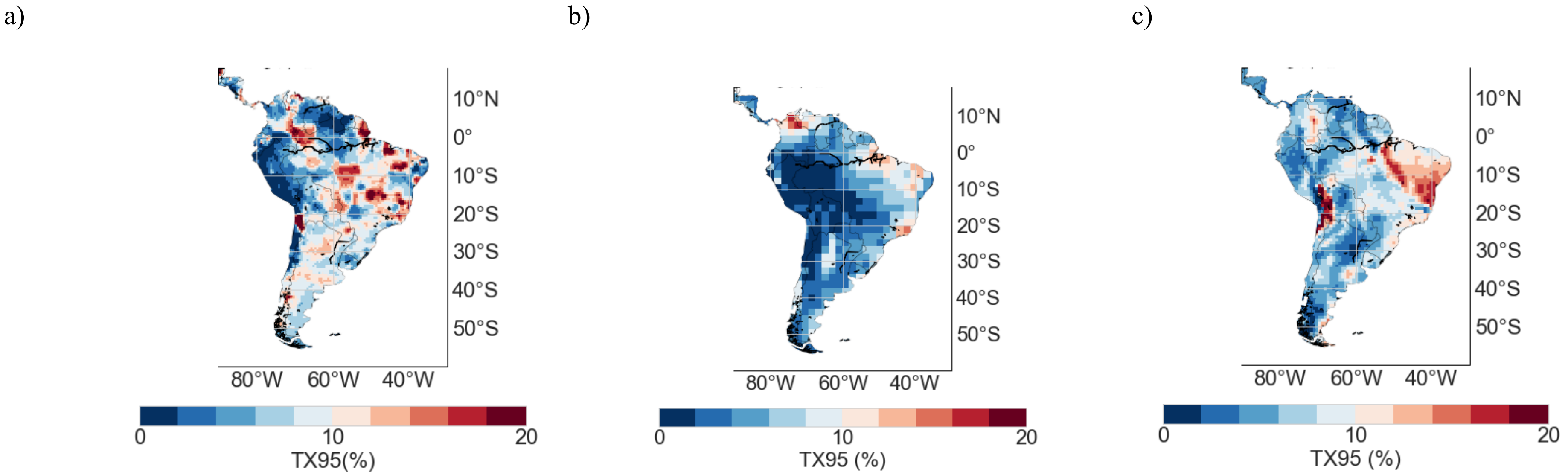

Fig. S4. TX95 estimates computed over the period 2006-2015 from different reanalysis datasets: a) the Climate Prediction Center (CPC) dataset; b) NCEP-DOE Reanalysis 2; c) ERA-Interim (ERAI). For the plots in this figure, the base period 1979-2005 was used. Plots were generated by using PYTHON's Matplotlib Library[92].

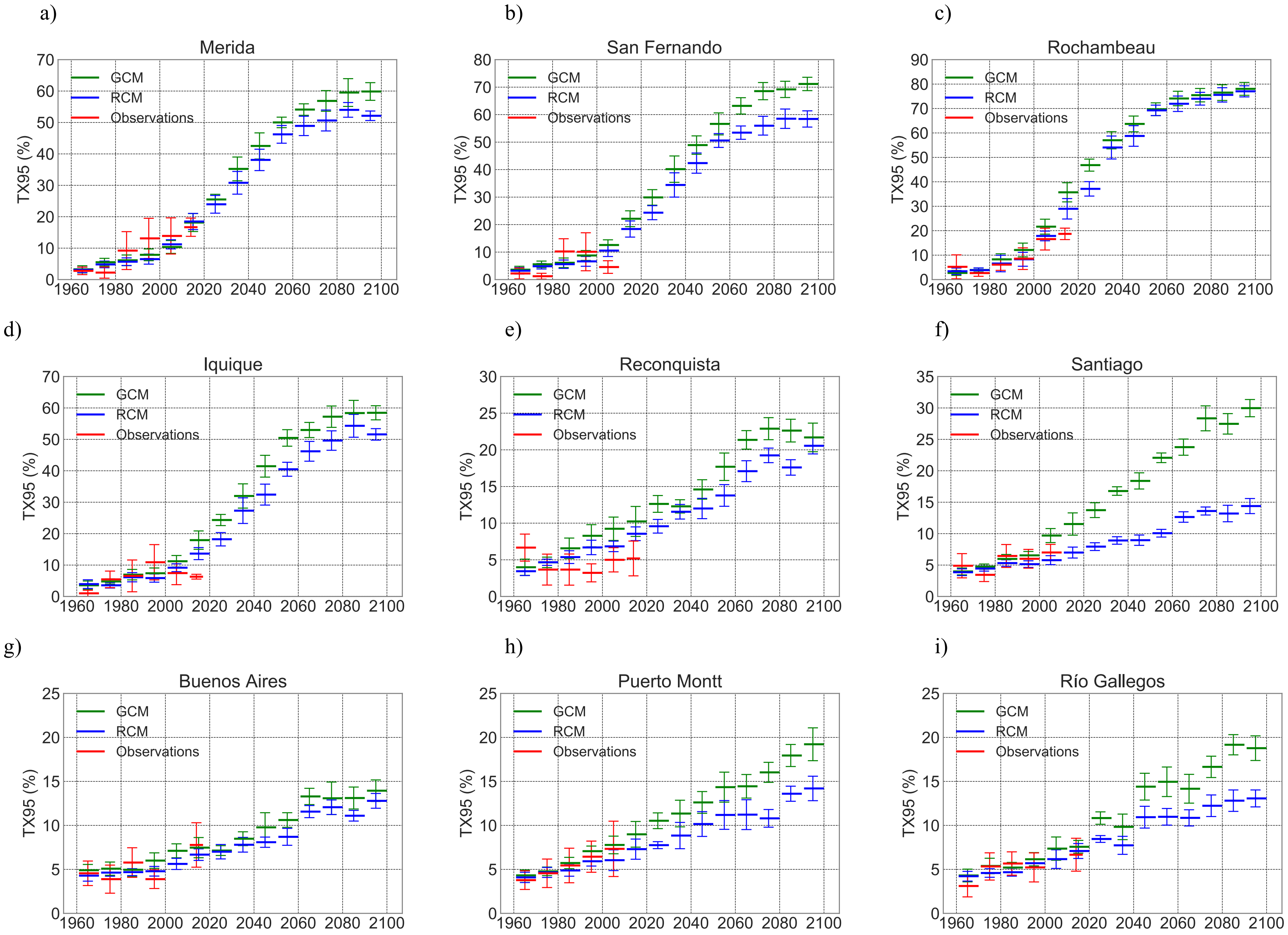

Fig. S5: Share of extremely warm DJF days (TX95) from observations over the period 1961-2016 (red lines), as well as from RCM simulations (blue lines) and from GCM data (green lines), over the period 1961-2005 and over the period 2006-2100 (under the RCP4.5 scenario). a) Merida; b) San Fernando; c) Rochambeau; d) Iquique; e) Reconquista; f) Santiago; g) Buenos Aires; h) Puerto Montt; i) Rio Gallegos. Error bars for each decade are based on the standard deviation computed by using annual TX95 estimates. Plots were generated by using PYTHON's Matplotlib Library[92].

Table S1: HW metrics and TX95 estimates over the base period 1961-1990, and over the period 2046-2055 (under the RCP4.5 scenario) for selected cities.

	**HWD [days]**		**HWF [days]**		**HWN**		**HWA [°C]**		**TX95 [%]**	
	**1961-1990**	**2046-2055**	**1961-1990**	**2046-2055**	**1961-1990**	**2046-2055**	**1961-1990**	**2046-2055**	**1961-1990**	**2046-2055**
**Bogotá**	6	19	2	44	0,5	5	1	3	5	54
**Buenos Aires**	4	4	1	3	0,3	1	3	4	5	9
**Caracas**	4	13	2	28	0,6	4	1	3	5	34
**Fortaleza**	10	33	3	46	0,5	3	1	2	5	52
**Guayaquil**	5	19	2	35	0,5	4	1	2	5	45
**Iquique**	5	14	3	31	0,6	4	1	2	5	36
**Lima**	6	16	2	32	0,5	4	1	2	5	42
**Mérida**	5	13	2	35	0,4	4	1	2	5	46
**Puerto Montt**	4	5	1	5	0,4	1	5	6	5	10
**Reconquista**	4	6	2	9	0,5	2	2	3	5	16
**Río Gallegos**	3	4	1	4	0,2	1	4	5	5	12
**Rochambeau**	9	43	3	61	0,5	3	1	2	5	70
**San Fernando**	6	24	3	42	0,6	4	1	2	5	51
**Santa Cruz**	5	7	2	13	0,5	3	2	3	5	21
**Santiago**	4	4	1	4	0,3	1	3	4	5	10
**Sao Paulo**	8	18	3	24	0,4	3	1	1	5	27
